# Warning Messages in Crisis Communication: Risk Appraisal and Warning Compliance in Severe Weather, Violent Acts, and the COVID-19 Pandemic

**DOI:** 10.3389/fpsyg.2021.557178

**Published:** 2021-04-01

**Authors:** Maxi Rahn, Samuel Tomczyk, Nathalie Schopp, Silke Schmidt

**Affiliations:** ^1^Department of Health and Prevention, University of Greifswald, Greifswald, Germany; ^2^Federal Office of Civil Protection and Disaster Assistance, Bonn, Germany

**Keywords:** COVID-19, severe weather, violent acts, warning message, risk appraisal, risk communication, warning compliance

## Abstract

**Background:**

In crisis communication, warning messages are key to informing and galvanizing the public to prevent or mitigate damage. Therefore, this study examines how risk appraisal and individual characteristics influence the intention to comply with behavioral recommendations of a warning message regarding three hazard types: the COVID-19 pandemic, violent acts, and severe weather.

**Methods:**

A cross-sectional survey examined 403 German participants from 18 to 89 years (*M* = 29.24; 72% female). Participants were allocated to one of three hazard types (COVID-19 pandemic, violent acts, severe weather) and presented with warning messages that were previously issued via an official warning app. Four components of risk appraisal—perceived severity (PS), anticipated negative emotions (AE), anticipatory worry (AW), and risk perception (RP)—were assessed before and after presenting the warning message. Path models were calculated to predict the intention to comply with the warning message, controlling for age, gender, and previous hazard experience.

**Results:**

For the COVID-19 pandemic, higher age (β = 0.18) predicted warning compliance (*R*^2^ = 0.05). AE (β = 0.20) predicted compliance in the case of violent acts (*R*^2^ = 0.09). For severe weather, PS (β = 0.28), age (β = 0.29), and female gender (β = 0.34) lead to higher compliance (*R*^2^ = 0.27). Changes across risk appraisal components were not consistent, as some facets decreased after the receipt of a warning message.

**Discussion:**

Risk appraisal has shown a marginal yet differential influence on warning message compliance in different types of hazards. Regarding the COVID-19 pandemic, the impact of sociodemographic factors on compliance should be studied more intensively. Moreover, integrating intermediary variables, such as self-efficacy, is necessary.

## Introduction

Crisis communication aims to inform the public about various kinds of impending threats and hazards. Warning messages are a means of communicating risks and giving advice on how to act correctly in case of such hazards (Mileti and Peek, 2000; Mayhorn and McLaughlin, 2014). This is as important for everyday perils as it is for new or still unknown threats and crises.

The outbreak of the novel coronavirus COVID-19 in 2019 and the ongoing pandemic pose new challenges in this respect: At the end of April 2020, more than 2.5 million people have become infected with this respiratory disease, and more than 180,000 thousand died (World Health Organization, 2020b). At this point in time, further development seemed yet unclear, as various factors were still unknown. Challenges arose, for example, from an uncertain case fatality rate, duration of infectiousness, pre-symptomatic infectiousness, as well as asymptomatic courses (Anderson et al., 2020; Peeri et al., 2020).

To flatten the pandemic curve, a quick adaption to this new threat is necessary. This means *inter alia* that the public at large must be provided with information and recommendations for protective measures, as effective vaccination is not yet available. Therefore, the World Health Organization (2020a) gives a series of advice for the public to control the further spread of COVID-19. Among others, recommendations include maintaining social distance, respiratory hygiene, washing hands, and not touching eyes, nose, and mouth as well as following advice given by healthcare providers and public health services. For these measures to be effective, they must be shared with as many people as possible. Moreover, they must also be implemented and complied with by the public. Warning messages, again, are essential for this purpose.

To construct effective warning messages, several factors must be considered. In addition to characteristics of the warning message itself, these include contextual factors, such as the communication channel, as well as characteristics and processes on the receiver’s side (Mileti and Sorensen, 1990; Mayhorn and McLaughlin, 2014; Bean et al., 2015). Among other theoretical frameworks addressing such processes, the Protective Action Decision Model (PADM) focuses on human responses toward threats (Lindell and Perry, 2012). According to the PADM, warning messages as well as contextual cues can initiate pre-decisional processes (exposure, attention, and comprehension of the cue or warning message) that, in turn, influence three core perceptions, namely perceptions of risk or threat, possible protective actions, and stakeholder perceptions. These pre-decisional processes and core perceptions are key to decision-making for those at risk. Characteristics of the warning message receiver, his or her channel access, and channel preference, as well as the source of the incoming information, are also considered in the PADM. In light of this theoretical background, warning messages can start multi-stage processes by communicating risk and giving recommendations on protective actions, with the appraisal of risk being pivotal.

### Risk Appraisal and Information Processing

Risk perception can be defined as a person’s beliefs about the vulnerability toward experiencing a potential threat. It is often operationalized as a subjective judgment of likelihood and thus conceived as a cognitive appraisal. Though, beyond this cognitive conceptualization, risk appraisal as well includes affective components that address feelings associated with a threat, for example, fear, sadness, or anger (Slovic, 1987; Sheeran et al., 2014). Previous research on risk appraisal toward threats and hazards, for instance, SARS or the avian flu (Leppin and Aro, 2009; Sheeran et al., 2014), points to a broad variation in conceptualization and assessment. This applies as well for natural hazards (Wachinger et al., 2013) or health-related behaviors (Sheeran et al., 2014), making it difficult to derive consistent conclusions and compare findings across scenarios, situations, and settings.

Seminal theoretical frameworks have focused either on the role of cognitions or affect toward risk and related attitudes and behaviors, such as the extended parallel process model (Witte, 1994; Popova, 2012), and the affect heuristic (Finucane et al., 2000; Slovic and Peters, 2006; Slovic et al., 2007). According to the extended parallel process model, cognitive threat appraisal as well as efficacy appraisal influence the likelihood of considering protective action, whereas affective appraisal (i.e., dread) can also lead to maladaptive behaviors, namely fear control, if efficacy is perceived as low. The affect heuristic focuses on the impact of affect and illustrates decision-making in high pressure situations. Consequently, under threat, negative affect activates the experiential system (i.e., automatic, intuitive information processing) that fosters swift action toward survival. The analytic system, on the other hand, represents a slower and more effortful way of processing information that is connected to information seeking, actively weighing pros, and cons before performing behaviors. Regarding disaster scenarios, analytic processing is likely if one has enough lead time to seek and process further information, prior experience, and knowledge of the disaster and protective behaviors. If information and lead time are scarce, experiential processing is more likely. Thus, depending on the situation, both cognitive and affective risk appraisals are important to compliance. This reasoning is echoed by research on health behaviors: A meta-analysis found that heightening and combining cognitive and affective appraisals of risk appraisal increases the intention to act and behavior itself (Sheeran et al., 2014).

For the ongoing COVID-19 pandemic, findings on risk appraisal and the adoption of protective measures are still preliminary. In a Hong Kong population at the beginning of the outbreak, participants of a survey reported high perceived susceptibility and high perceived severity toward COVID-19. In contrast, the willingness to distance oneself socially in the sample varied, with 39%–88% intending to take this action (Kwok et al., 2020). In another study with a US American sample, risk perception (assessed as infection likelihood and severity for oneself and others) increased during the first week of the pandemic in northern America, while participants’ risk perception was higher for others than for themselves. In this sample, the adoption of protective measures, such as social distancing and hand-washing, increased as well during this first week, with protective measures being predicted by the perceived likelihood of becoming infected (Wise et al., 2020). Moreover, the role of individual characteristics on the appraisal of risk and the adoption of protective measures becomes apparent: In a German population, younger age was associated with a higher perceived likelihood of becoming infected by COVID-19 (for self, others, and in general), while females and the elderly worried more about becoming infected (Gerhold, 2020). Also, female gender and higher subjective knowledge of COVID-19 made it more likely for Hong Kong inhabitants to socially distance (Kwok et al., 2020). Findings on prior pandemics show a similar picture: For several infectious diseases, such as avian influenza, SARS, or swine influenza, older age, female gender, higher education, being non-white, as well as perceived susceptibility and severity of the respective disease predicted the adoption of protective measures (Bish and Michie, 2010).

To provide context for the analysis of warning communication in the COVID-19 pandemic, the present study aims to compare the ongoing pandemic to two additional hazard types, namely severe weather and violent acts, with varying degrees of severity and familiarity. On the one hand, severe weather, such as thunderstorms or heavy rainfalls, is a familiar event in Germany and mostly characterized by moderate severity, with yet increasing economic damages (Coronese et al., 2019). Violent acts, on the other hand, are comparatively rare but of tremendous impact (Sheppard, 2011). That a comparative approach might be useful is shown by a broad body of research that focuses on specific hazards or singular events only while assessing risk appraisal inconsistently. Also, to our knowledge, only a few studies aim to compare different hazards in terms of risk appraisal. Their findings show that various hazard types are perceived differently in terms of risk appraisal (Rahn et al., 2020) and vary in how likely protective measures are intended, for example, due to a variation in threat imminence or risk level (Ho et al., 2008; Heilbrun et al., 2010). The type of hazard as well influences cognitive and affective components of risk appraisal when receiving warning messages, including interactional effects of hazard type and characteristics of the message receiver (Rahn et al., 2020). Consequently, it is of interest whether the different components of risk appraisal influence the compliance of the protective measures and whether the hazard types differ in this respect.

Severe weather (e.g., thunderstorms, lightning, heavy rain- or snowfall) is experienced frequently by the public. This hazard type can be subsumed as a natural hazard, for which previous experience is a factor that is associated with the perception of risk (Ho et al., 2008; Olofsson and Rashid, 2011; Wachinger et al., 2013; Frondel et al., 2017). Despite a broad body of research, the relationship between risk appraisal, previous experience, and the adoption of protective measures is inconclusive, as findings are inconsistent and additional factors, as well as complex pathways, were found (Wachinger et al., 2013). Yet, warning messages regarding severe weather can lead to faster adoption of protective measures, for example, in the event of a thunderstorm (Markwart et al., 2019). In the present study, we used a warning message addressing a thunderstorm with chances of lightning and storm.

In contrast to severe weather, violent acts are experienced less likely, while being fairly severe, and therefore serving as an upper limit when comparing risk appraisal. In this study, violent acts are defined as directed, mostly planned acts of violence against people, which usually occur unexpectedly and cause deaths or injuries. In the early onset of a violent act, it is often unclear whether it is a rampage, terrorist threat, or any other kind of assault. For terror threats, risk perceptions toward terror as well as sociodemographic factors are associated with the anticipated emergency response (Gibson et al., 2015). Individual characteristics, such as trait anxiety, as well as perceptions of vulnerability and self-efficacy, were found to be associated with preparedness behavior in terror threats, too (Wirtz et al., 2019). Again, in the case of violent acts of all kinds, warning messages are a key to providing the public with information in near real-time (Reuter et al., 2017). The warning message used in this study addressed a rampage in a city center, with a still unknown number of active shooters on the run. In this case, a violent incident had already occurred, so that a warning was indispensable. However, the exact outcome (number of deaths or injuries, unclear number of suspects) and the background of the violent act were still unknown at the time when the public received the warning message.

The influence of risk appraisal on warning message compliance regarding different types of hazards seems unclear. This applies especially to the new COVID-19 pandemic. Moreover, individual characteristics of those at risk, such as previous experience with a hazard or sociodemographic factors, play an important role in risk appraisal and the adoption of protective measures. While controlling for characteristics on the receiver’s side, the present study aims to explore the links between cognitive and affective components of risk appraisal on the intention to comply with protective measures given in a warning message. Moreover, these interrelations are examined for three different types of hazards, namely severe weather, violent acts, and the COVID-19 pandemic.

## Materials and Methods

The present study was approved by the ethical committee of the University Medicine of Greifswald (BB 169/18) and included informed consent in alignment with the Declaration of Helsinki.

### Sample

Participants were recruited via internet forum posts and flyer advertising. As incentives, they were offered 5 € or a voucher of the same value as compensation of expense. Data was collected online (questionnaire via hyperlink) and offline (via paper-pencil questionnaire) for severe weather and violent acts. For COVID-19, data collection took place online only.

For severe weather and violent acts, a subsample was collected during a period of eight months from May to December 2019. Data collection regarding the COVID-19 pandemic took part between March 13 and March 27, 2020. The latter period covers the beginning of the COVID-19 outbreak in Germany and the start of large-scale measures by the German government, such as social distancing and closing of public institutions.

### Materials

Participants were presented warning messages that had been previously used to warn the German public of severe weather, a violent act, and the COVID-19 pandemic. Because of that, wording, content, and sender of the warning message were already fixed. The warning messages were staged into the format of a warning application for smartphones, called NINA (BBK, 2020). NINA is free of charge for the public and provided by the Federal Office of Civil Protection and Disaster Assistance. It is used by the German government, federal states, and local communities to provide location-based warning messages via push notifications. Hazards to which the app refers include threatening weather situations as well as large-scale emergencies and national or local threats (Petridou et al., 2019).

Before receiving the warning messages, participants received a short description of the hazard, which was presented in German:

– Severe weather: Severe weather is an umbrella term referring to different weather-related events. Severe weather can have immense consequences and threaten public safety. Among others, severe weather comprises heavy rain, severe storms, thunderstorms, or extreme snow.
– Violent acts: Violent acts are targeted, mostly planned acts of violence against people, which usually occur unexpectedly. Often people are injured or killed.
– COVID-19 pandemic: Coronaviruses cause a variety of diseases in humans, ranging from common colds to dangerous or even potentially fatal diseases. The novel coronavirus (COVID-19) is transmissible from person to person. The main mode of transmission is droplet infection.

All warning messages included information about the particular hazard, as well as recommendations for action. The warning message regarding severe weather referred to a heavy thunderstorm with possible lightning and storms. The message on the violent act warns about a yet unknown violent incident in the center of a city, with suspects still on the run. The message regarding COVID-19 as well consisted of information about COVID-19 (e.g., number of cases confirmed to date and action taken by the authorities) and recommendations for action to prevent an infection. The latter warning message was used in March 2020 in a district of Northern Germany.

For severe weather and violent act, warning messages (including English translations) can be found elsewhere (Rahn et al., 2020). The English translation of the warning on COVID-19 is provided in the supplementary.

### Measures

Sociodemographic data included age, gender (1, female; 2, male), and previous experience with severe weather, violent acts, or pandemics. For previous hazard experience, participants were asked whether they or a person close to them (e.g., family, friends) had ever experienced the hazard. Experience was given, when one of these questions was answered with “yes” (0, no previous experience; 1, previous experience).

For the assessment of risk appraisal, a facetted approach was chosen in this study, measuring risk with four components: (1) perceived severity (PS), (2) anticipated negative emotions (AE), (3) anticipatory worry (AW), and (4) risk perception (RP) (Sheeran et al., 2014). PS and RP are considered cognitive facets, while AE and AW are considered affective components of risk appraisal (Leppin and Aro, 2009; Sheeran et al., 2014). Risk appraisal was assessed at two points in time, before (1) and after (2) the receipt of a warning message regarding one out of three hazards. For PS (“How serious would the consequences be for you if __________ happened?”), AE (“How would you feel if __________ happened?” [anxious, tense, sad]), and AW (“How worried are you that you might be affected by __________?”), five-point Likert scales were used ranging from 1 (*not at all*) to 5 (*very much*). For AE, mean values of the three negative emotions were calculated, showing good internal consistency (Cronbach’s α = 0.82–0.84). RP (“How likely is it that you could be affected in the future by _________?”) was assessed via visual analog scale ranging from 0% to 100%.

To assess the intention to act, the participants were asked how likely they would follow with the particular recommendations given in the warning message using five-point Likert scales (1, *not at all* to 5, *very much*). For each participant, a mean value was calculated for the intention to act. Protective measures for the three hazard types included:

– Severe weather (four recommendations): Close windows and doors; secure objects outdoors; keep away from buildings, trees, scaffolding, and power lines; avoid staying outside.
– Violent acts (four recommendations): Avoid streets and public places; turn on radio and television; stay at home; share the warning message.
– COVID-19 (nine recommendations): Cover mouth and nose with elbow or tissue when coughing; not shaking hands; avoid touching eyes, nose, and mouth; use and safe disposal of used tissues; intensive room ventilation; maintain hand hygiene; stay at home in case of illness/symptomatic; avoid contact with possibly ill persons; avoid mass events.

### Design and Study Procedure

A cross-sectional survey design was conducted. All participants received study information and stated their informed consent before starting the survey. For severe weather and violent acts, participants were randomly allocated to one of the two disaster types. To avoid ambiguity, participants received a short explanation of their hazard type. After that, previous experience and the four components of risk appraisal were assessed. Participants then received a warning message with the instruction to imagine that they were affected by the hazard described therein. Lastly, risk appraisal was assessed again, as well as the intention to comply with the specific recommendations given in the presented warning message.

### Statistics

**FIGURE 1:**
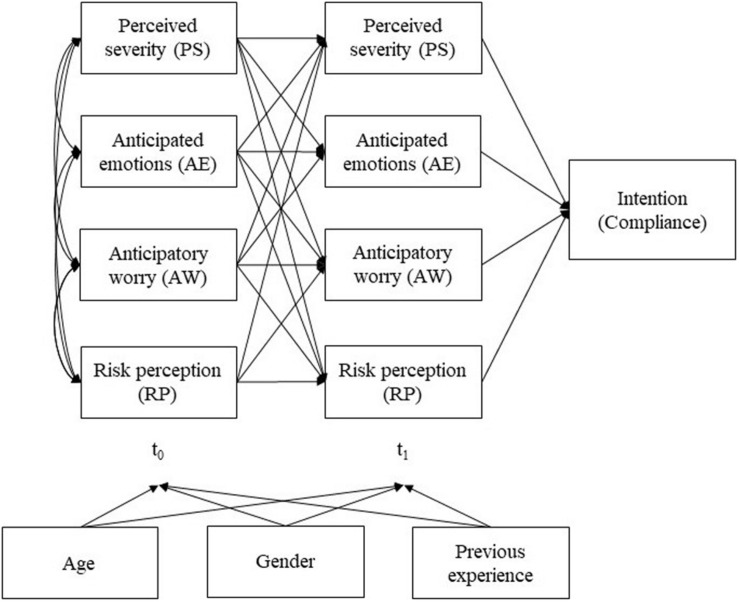
Simplified path model. Four components of risk appraisal (PS, AE, AW, RP) before (*t*_0_) and after (*t*_1_) the receipt of a warning message regarding one of three hazard types (severe weather, violent act, or the COVID-19 pandemic).

IBM SPSS 25 and IBM Amos 25 were used for the statistical analyses. First, tests were conducted to investigate the links between hazard type and age (univariate ANOVA), previous experience, and gender (Chi-square tests). Bivariate (Pearson) correlations were then used to explore associations between all examined variables. To examine the influence of all four components of risk appraisal combined on warning message compliance, path models were calculated for each hazard type, controlling for age, gender, and previous hazard experience. Path models were estimated using the Full Information Maximum Likelihood method in consideration of missing data (Enders, 2001) and calculated without and with the control variables. A simplified path model for all three hazards is shown in Figure 1.

## Results

Descriptive statistics can be found in Table 1, with pairwise correlations in Table 2. A total of 403 adults (*M* [SD]_age_ = 29.24 [13.99], 72.2% female) took part in the survey. In total, 33.5% of the participants had previous hazard experience, ranging from 7.7% (pandemics) to 77.2% (severe weather). Participants allocated to the three hazard types did not differ by age [*F*(2, 402) = 2.23, *p* = 0.109]. Thus, they did differ by gender [*χ*^2^(2) = 7.68, *p* = 0.021] and previous experience [*χ*^2^(2) = 158.69, *p* = 0.001]. The latter seems reasonable, as severe weather is experienced far more often than violent acts or pandemics.

**TABLE 1:** Descriptive statistics of age, gender, previous hazard experience, the components of risk appraisal before (1) and after (2) the receipt of a warning message, and the intention to comply with a warning message, displayed for the complete sample and separated by hazard type.

		Hazard type		
	Complete sample	Severe weather	Violent act	COVID-19
	*N* = 403	*n* = 123	*n* = 125	*n* = 155
**Gender**				
% male	27.8 (112)	33.3 (41)	32.0 (40)	20.0 (31)
% female	72.2 (291)	66.7 (82)	68.0 (85)	80.0 (124)
Previous experience (% yes)	33.5 (135)	77.2 (95)	22.4 (28)	7.7 (12)

	***M* (SD)**	***M* (SD)**	***M* (SD)**	***M* (SD)**

Age	29.24 (13.99)	31.28 (15.73)	29.10 (15.32)	27.73 (10.98)
**Risk appraisal**				
Perceived severity 1	3.05 (1.12)	2.84 (1.00)	3.86 (1.00)	2.59 (0.95)
Perceived severity 2	3.01 (1.14)	2.76 (1.01)	3.78 (1.07)	2.60 (0.98)
Anticipated emotions 1	2.95 (1.07)	2.35 (0.83)	3.85 (0.91)	2.71 (0.87)
Anticipated emotions 2	2.84 (1.10)	2.26 (0.84)	3.79 (0.90)	2.54 (0.93)
Anticipatory worry 1	2.56 (1.11)	2.40 (1.03)	2.26 (1.10)	2.93 (1.09)
Anticipatory worry 2	2.57 (1.05)	2.55 (0.98)	2.45 (1.15)	2.68 (1.01)
Risk perception 1	50.99 (27.01)	55.78 (27.02)	34.10 (24.78)	61.40 (21.52)
Risk perception 2	52.33 (26.76)	58.51 (26.96)	36.09 (24.51)	59.97 (22.60)
Intention to comply	4.33 (0.66)	4.13 (0.85)	4.33 (0.69)	4.49 (0.36)

*M, mean; SD, standard deviation. Risk appraisal measured before (1) and after (2) the receipt of a warning message including the components perceived severity, anticipated emotions, anticipatory worry (five-point Likert scales from 1, *not at all* to 5, *very much*), and risk perception (%).*

**TABLE 2:** Pairwise (Pearson) correlations of age, gender, previous experience, risk appraisal before (1) and after (2) the receipt of a warning message, and the intention to comply, *N* = 377–403.

		1	2	3	4	5	6	7	8	9	10	11	12
1	Age	1											
2	Gender	−0.21***	1										
3	Previous experience	0.05	−0.06	1									
4	Perceived severity 1	0.17**	0.03	−0.05	1								
5	Perceived severity 2	0.16**	0.02	−0.05	0.83***	1							
6	Anticipated emotions 1	0.06	0.14**	−0.19***	0.61***	0.60***	1						
7	Anticipated emotions 2	0.05	0.15**	−0.14**	0.60***	0.61***	0.91***	1					
8	Anticipatory worry 1	0.06	0.12*	−0.05	0.24***	0.32***	0.28***	0.25***	1				
9	Anticipatory worry 2	0.10	0.19***	0.02	0.37***	0.48***	0.34***	0.36***	0.69***	1			
10	Risk perception 1	−0.07	0.16**	0.15**	−0.24***	−0.16**	−0.28***	−0.29***	0.33***	0.21***	1		
11	Risk perception 2	−0.11*	0.21***	0.20***	−0.20***	−0.15**	−0.25***	−0.25***	0.29***	0.27***	0.89**	1	
12	Compliance	0.16**	0.20***	−0.14**	0.17**	0.13**	0.17**	0.15**	0.17**	0.15**	0.08	0.06	1

Gender (1, male; 2, female); previous experience (0, no previous experience; 1, previous experience given). Risk appraisal before (1) and after (2) the receipt of a warning message: Perceived severity, anticipated emotions, and anticipated worry were assessed on five-point Likert scales from 1 (*not at all*) to 5 (*very much*); risk perception was assessed in %. *p < 0.05, **p < 0.01, ***p < 0.001.

Bivariate correlations showed significant positive associations of all variables with the intention to comply with the warning message, except for previous experience (*r* = −0.14, *p* < 0.01), RP1, and RP2 (*r* = 0.06–0.08, *p* > 0.05). Also, positive correlations were found for age, PS1, and PS2 (*r* = 0.16–0.17, *p* < 0.01) as well as age and RP2 (*r* = −0.11, *p* < 0.05). For gender, positive correlations were found for all components of risk appraisal, except for PS1 and PS2. For previous experience, significant positive (RP1, RP2) and negative (AE1, AE2) correlations were found.

Interestingly, there was no consistent trend in the change of risk appraisal after the receipt of a warning message. Some components decreased while others increased: For COVID-19, AW (*M*_AW__1_ = 2.93; *M*_AW__2_ = 2.68) and RP (*M*_RP__1_ = 61.40; *M*_RP__2_ = 59.97) decreased, while severe weather and violent acts showed an increase after the receipt. In contrast to that, AE decreased in all hazard types.

Path models for severe weather, violent acts, and the COVID-19 pandemic can be found in Table 3. For the three types of hazards, path models including all covariates (model 2) revealed different factors that had a direct influence on the intention to comply with the warning message, while showing good to moderate model fits.

**TABLE 3:** Path models for the hazard types severe weather, violent act, and COVID-19, with (model 2) and without age, gender, and previous experience (model 1) as covariates.

	Severe weather		Violent act		COVID-19	
	Model 1	Model 2	Model 1	Model 2	Model 1	Model 2
Chi squared test (goodness-of-fit test)	3.203 (df = 6)	6.805 (df = 6)	11.533 (df = 6)	10.559 (df = 6)	6.151 (df = 6)	6.735 (df = 6)
CFI	1.000	0.999	0.992	0.994	1.000	0.999
TLI	1.000	0.984	0.943	0.922	0.999	0.990
RMSEA	0.000 [0.000;0.078]	0.033 [0.000;0.125]	0.086 [0.000;0.160]	0.078 [0.000;0.154]	0.013 [0.000;0.105]	0.028 [0.000;0.111]
**Variables**						
RP1 → RP2	0.883***	0.848***	0.908***	0.880***	0.814***	0.801***
AW1 → AW2	0.416***	0.421***	0.702***	0.709***	0.388***	0.384***
AE1 → AE2	0.709***	0.705***	0.879***	0.865***	0.916***	0.911***
PS1 → PS2	0.532***	0.513***	0.625***	0.642***	0.852***	0.828***
RP2 → compliance	0.141	0.122	0.011	0.025	0.043	0.052
AW2 → compliance	0.065	−0.068	−0.052	−0.058	0.090	0.083
AE2 → compliance	0.013	0.016	0.253*	0.200*	0.106	0.097
PS2 → compliance	0.249	0.279*	0.005	0.003	−0.102	−0.153
Age → compliance	–	0.292***	–	0.160	–	0.176*
Gender → compliance	–	0.338***	–	0.123	–	0.060
Previous experience → compliance	–	0.004	–	−0.059	–	0.014
*R*^2^ (compliance)	0.126	0.265	0.059	0.091	0.021	0.051

Standardized model results; * *p* < 0.05, ** *p* < 0.01, *** *p* < 0.001; brackets indicate 90% confidence interval. CFI, comparative fit index; TLI, Tucker-Lewis index; RMSEA, root mean square error of approximation. Components of risk appraisal: RP, risk perception; AW, anticipatory worry; AE, anticipated emotions; PS, perceived severity. Risk appraisal was measured at two time points, before (1) and after (2) receiving a warning message.

For severe weather, the path model showed a significant influence of PS (β = 0.28), higher age (β = 0.29), and female gender (β = 0.34) on the intention to comply with the recommendations given in the warning message (*R*^2^ = 0.27; CFI = 0.999; TLI = 0.984; RMSEA = 0.033).

For violent acts, AE (β = 0.20) predicted the intention to comply (*R*^2^ = 0.09; CFI = 0.994; TLI = 0.922; RMSEA = 0.078).

For the COVID-19 pandemic, higher age (β = 0.18) predicted warning compliance (*R*^2^ = 0.05; CFI = 0.999; TLI = 0.990; RMSEA = 0.028).

## Discussion

The present study examined warning message receipt, risk appraisal, and the intention to comply with a warning message while applying a consistent methodology in assessing risk appraisal with two cognitive and two affective components. Additionally, three types of hazards were compared: severe weather, violent act, and the COVID-19 pandemic. Sociodemographic factors were taken into account as well. As seen in preceding research (Ho et al., 2008; Heilbrun et al., 2010; Rahn et al., 2020), heterogeneous results between the hazard types were found.

For severe weather, perceived severity (PS) led to a higher intention to comply with the warning message. The more severe the hazard is perceived, the more likely it is to carry out the recommendations. This finding is consistent with the theoretical assumption of the PADM, as the perception of the impending threat and its severity play an important role in the adoption of protective measures (Lindell and Perry, 2012). Besides, in the event of a thunderstorm, in most cases, it is possible to prepare for the hazard for a certain period. The pros and cons of implementing protective measures can be considered. This time lead could result in an analytical processing and, in turn, the cognitive component of risk appraisal influencing the intention to act. Also, higher age and female gender were associated with warning message compliance. This goes with prior research that found an association between age and female gender regarding warning message response, and the likelihood of seeking shelter in the case of severe weather or tornados (Ryherd, 2016). Other findings show that persons over 35 years show a better understanding of warning messages regarding weather events, report a better understanding of possible outcomes, and report a higher concern toward the event as well as higher intention to adopt protective measures (Potter et al., 2018).

Looking at violent acts, an influence of anticipated negative emotions (AE) on warning message compliance was found: The more anxious, tense, or sad participants felt about becoming involved in a violent act, the more likely it was for them to comply with the warning message. The occurrence of violent acts is associated with high potential threat and to some point unknown consequences for the ones involved. This may have an influence on which processes they cause in individuals when becoming confronted with a warning message regarding a violent act. Media coverage of terror threats, for example, has been shown to induce fear (Slone, 2000). According to the affect heuristic, affective reactions toward stimuli (e.g., a feeling state of badness toward violent acts) influence judgments, decisions, and behavior. These so-called affect-based evaluations appear to happen quickly and are therefore mostly applied under time pressure, as they are processed through the experiential system (Finucane et al., 2000; Slovic et al., 2007). The warning message used for this study was issued during a violent act in a German city. It comprised a short text about a somewhat unknown and rare threat and was not able to determine full information of the exact nature of the hazard (rampage, terror threat, or else) at the same time. Additionally, the protective measures given in the message required a prompt reaction. In line with the affect heuristic, this could promote an experiential processing of the warning message, which, in turn, could be a possible explanation of the identified link between an affective component of risk appraisal, namely anticipated negative emotions, and the intention to comply in case of violent acts (Lerner et al., 2003). In contrast to severe weather and violent acts, no relationship between risk appraisal and the intention to comply with the warning message was found for COVID-19. These results seem to be in line with other research that also found little or no impact of risk perception on compliance regarding COVID-19 (Clark et al., 2020). Pandemics could be perceived as more controllable than violent acts, for instance, as there are a variety of protective measures for this hazard that can be consciously integrated into everyday life. In this context, the usage of protective measures can lead to risk appraisal being nullified or reduced, as if someone already carries them out, perhaps he or she will appraise the risk of becoming infected lower (Brewer et al., 2004; Leppin and Aro, 2009). Thus, this could turn pandemics into special cases. Ongoing investigations should address whether this also applies to the COVID-19 pandemic as well, as changes in risk appraisal seem to be possible in the further development of this pandemic. Yet, when looking at the covariates, a higher age was a significant predictor for the intention to comply. The latter finding is consistent with prior results regarding the beginning of the COVID-19 pandemic (Tomczyk et al., 2020) as well as other infectious diseases (Bish and Michie, 2010). In the COVID-19 pandemic, persons with a higher age were considered a high-risk group from the very beginning, for example, due to more severe disease progression and higher mortality (Bhopal and Bhopal, 2020; Kang and Jung, 2020). This might result in a higher appraisal of risk, particularly perceived susceptibility, among older persons, which in turn could lead to the adoption of protective measures (Barr et al., 2008).

In the comparison of the three hazard types, no consistent influence of age, gender, and previous experience on the intention to comply with a warning message were found. This applies as well for risk appraisal and the intention to comply: While a cognitive component of risk appraisal showed an influence on warning message compliance in the case of severe weather, affective appraisal seemed to predict the intention to comply in case of violent acts. Besides, no trend in the changes of risk appraisal after the receipt of a warning message was found, as for some hazard types components increased while others decreased. For violent acts and severe weather, the trend after receiving the warning is a slight decrease in perceived severity and anticipated negative emotions. Despite these being marginal changes, participants seemed to rate these hazards as less severe and have less negative emotions toward them when receiving a warning message. On the other hand, risk perception and anticipatory worry increased. By issuing a warning message including protective measures, people at risk could develop a feeling of preparedness, which, in turn, could result in fewer negative emotions and the feeling of the hazard being less severe for oneself. Risk perception, here assessed as the probability of becoming affected by the hazard, and worry of becoming affected may increase due to the confrontation with a possible threat that requires a rather fast response. For COVID-19, a slightly different image becomes apparent: For almost every component, a decrease can be observed. Participants felt fewer negative emotions, less worry, and less susceptible after the receipt. As already mentioned above, pandemics could constitute an exception in this context since warning messages could deliver a feeling of security by giving sufficient information and enough time for the implementation of protective measures. Additionally, data collection took place at a very early stage of the pandemic in Germany. At this time, the COVID-19 pandemic had just reached Germany, and in some areas of the country, there were only a few or no cases. Besides, pandemics of this extent are rather rare in Central Europe, so that there was hardly any contact or previous experience with this topic before. The collection of data in the further course of this pandemic can bring exclusion here and is therefore desirable.

In summary, the given results lead to the point that risk appraisal should be assessed with both cognitive and affective components. Also, it becomes clear that findings in warning research regarding different hazard types cannot be transferred straightforwardly, as there are indications for varying processing. Especially concerning the COVID-19 pandemic, future research on risk appraisal and warning compliance should look at already existing research on other hazard types in a comparative rather than a separate way.

### Limitations

The present study certainly has some limitations: Our research aimed to compare three different hazards that varied in terms of several characteristics (e.g., frequency, extent of damage, proximity). Original, but anonymized, warning messages were presented to the participants. These warning messages had already been used to warn the public in Germany and, therefore, the content and design of the messages were not varied. Yet, empirical research shows that a variation of hazard characteristics, such as proximity of the hazard source, influences perceived risk, for example, in hurricanes, chemical hazards, and floods (Zhang et al., 2010). The psychometric paradigm (Slovic et al., 1986; Marris et al., 1997), according to which risk perception is influenced by common risk characteristics, such as controllability, dread, and knowledge of different hazards, could provide an additional perspective. On the other hand, the usage of original warnings in this study leads to a higher ecological validity of the presented results. Yet, future research should proceed with the use of authentic warning messages and also aim toward a systematical variation of the messages. Regarding the COVID-19 pandemic, the collected data only show a small part of a complex and fast process. Like others, we aimed to capture this process at an early stage, namely at the beginning of the restrictions in Germany. Further research must continue to collect data repeatedly in order to be able to make statements in the long term. This way, for example, a change in cognitive and affective appraisals of risk over time, as well as a change in behavioral intention and the adoption of protective measures, can be unveiled (Leppin and Aro, 2009). By doing so, upcoming studies should examine representative samples, as the presented findings are based on a convenience sample.

Also, further research should focus on additional variables that are included in the PADM, such as stakeholder perceptions or social norms, to improve the understanding of the link between risk appraisal and behavior. In the context of health-related behaviors, self-efficacy and response efficacy were shown to play important roles in the association between risk appraisal and behavioral intention or behavior (Sheeran et al., 2014) and should thus be considered for civil protection as well. This applies as well on the assessment of protective measures carried out by the public, as this study was scenario-based and therefore only able to assess behavioral intention. Nevertheless, recent research shows that experimental studies (in the sense of scenario-based studies) and field studies are equally suitable for the investigation of warning message understanding and response (Weyrich et al., 2018).

## Data Availability Statement

The raw data supporting the conclusions of this article will be made available by the authors, without undue reservation.

## Ethics Statement

The studies involving human participants were reviewed and approved by the Ethics Committee of the University Medicine of Greifswald. The participants provided their written informed consent to participate.

## Author Contributions

MR, ST, and SS: conception and design of the study. NC: preparation of the warning messages for severe weather and violent acts. MR and ST: collection of data. MR: organized the database and writing the first draft of the manuscript. ST: calculating path models. All authors contributed to the article and approved the submitted version.

## Conflict of Interest

The authors declare that the research was conducted in the absence of any commercial or financial relationships that could be construed as a potential conflict of interest.
