# Supplementary material for: Warning Messages in Crisis Communication: Risk Appraisal and Warning Compliance in Severe Weather, Violent Acts, and the COVID-19 Pandemic
Source: Front Psychol. 2021 Apr 1;12:557178. doi: 10.3389/fpsyg.2021.557178 (PMC8046918; doi:10.3389/fpsyg.2021.557178)
Supplement: Supplementary file 1 [file Table_1.DOCX]

**Supplementary**

English translation of the warning message used to warn the public about COVID-19.

**Warning:**

Fourth confirmed Corona infection in your state - Information from your federal ministry.

The Ministry of Health informs: After three known Corona infections in your state so far, there is a fourth laboratory-confirmed case. It concerns a man from the district of XX, who returned from Italy in the past few days and developed symptoms. The laboratory result from today confirmed the infection. The patient is in domestic quarantine, as are the identified contacts.

**Affected regions:**

Your state

**Information on protection against infection with Coronavirus**

The new Coronavirus is transmissible from person to person. As with influenza and other acute infections of the respiratory tract (so-called colds or flu-like infections), the following hygienic rules apply:

- adhere to the cough and sneeze etiquette, do not cough or sneeze on anyone, if no handkerchief is available, sneeze or cough into the crook of the elbow (not into the hands!)
- avoid shaking hands
- avoid touching the eyes, nose, or mouth
- use and safe disposal of disposable tissues
- intensive room ventilation
- exact hand hygiene in everyday life, e.g., thorough hand washing after contact with persons, after using sanitary facilities and before eating as well as after contact with objects or materials in public and after contact with sick persons
- recommending that people with the disease stay at home in their interest to prevent further infection
- if necessary, avoid large events

**Published by:**

Your state
